# Association between body mass index and suicidal behaviors: a systematic review protocol

**DOI:** 10.1186/s13643-015-0038-y

**Published:** 2015-04-19

**Authors:** Stefan Perera, Rebecca Eisen, Monica Bawor, Brittany Dennis, Russell de Souza, Lehana Thabane, Zainab Samaan

**Affiliations:** 1 Health Research Methodology Graduate Program, McMaster University, 1280 Main Street W, Hamilton, ON L8S 4L8 Canada; 2 Department of Clinical Epidemiology and Biostatistics, McMaster University, 1280 Main St. West, Hamilton, ON L8S 4L8 Canada; 3 MiNDS Neuroscience Graduate Program, McMaster University, 1280 Main Street W., Hamilton, ON L8S 4L8 Canada; 4 Population Genomics Program, Chanchlani Research Centre, McMaster University, 1280 Main St. West, Hamilton, ON L8S 4L8 Canada; 5 Biostatistics Unit, Centre for Evaluation of Medicine, 25 Main Street W. Suite 2000, Hamilton, ON L8P 1H1 Canada; 6 Department of Psychiatry and Behavioural Neurosciences, McMaster University, 1280 Main St. West, Hamilton, ON L8S 4L8 Canada

**Keywords:** Suicide, Attempted suicide, Suicidal ideation, Suicidal behavior, Body mass index, BMI, Obesity, Systematic review, Protocol

## Abstract

**Background:**

Suicide is among the leading causes of death worldwide. Suicide attempts and suicidal ideation are more common than completed suicide and are associated with psychological distress. These behaviors are considered risk factors of completed suicide. Considering the psychosocial stigma and medical comorbidities associated with obesity, an accumulating body of studies have investigated body mass index (BMI) as a potential risk factor of suicide. However, several cohort studies have demonstrated an inverse relationship between BMI and completed suicide, suggesting a protective effect of increasing BMI against completed suicide. The association between BMI and attempted suicide is more equivocal, with several studies reporting both positive and negative relationships between BMI and attempted suicide. The primary objective of this study is to systematically review the literature to determine the association between BMI and suicidal behavior (including completed suicide, attempted suicide, suicidal ideation) in an adult population (18 years and older). The secondary objective is to explore whether sex, age, and the method used in suicide modify the relationship between BMI and suicidal behavior.

**Methods/design:**

An electronic search will be conducted using PubMed/MEDLINE, PsycINFO, CINAHL, and EMBASE using a predefined search strategy; databases will be searched from their inception. Two authors (SP and RE) will independently screen articles using predefined inclusion and exclusion criteria and will extract pertinent data using a pilot tested extraction form. At all levels of screening, discrepancies between the two authors will be resolved by consensus, and in the case of disagreement, by consulting a third author (ZS). The primary outcomes include the association between BMI and completed suicide, attempted suicide, and suicidal ideation. If appropriate, a meta-analysis will be conducted. Risk of bias and quality of evidence will be assessed.

**Discussion:**

The results of this systematic review will inform health care professionals and researchers about whether BMI has a significant role in suicidal behavior and psychological well-being.

**Systematic review registration:**

PROSPERO CRD42014014739.

**Electronic supplementary material:**

The online version of this article (doi:10.1186/s13643-015-0038-y) contains supplementary material, which is available to authorized users.

## Background

Suicide is a significant problem throughout the world, impacting society from a public health, community, individual, and often a family perspective. Every year, over 800,000 lives are claimed by suicide [1]. Suicide is a complex phenomenon and requires evidence-based guidelines designed to recognize and mitigate modifiable suicide risk factors. A previous suicide attempt is considered a significant predictor of completed suicide in the general population [2,3]. Attempted suicide occurs 10 to 20 times more frequently than completed suicide [4]. Suicidal behaviors entail a complex set of ideas, thoughts, plans, and actions, which imply intention to end one’s life [4]. There are several well-established risk factors that collectively affect an individual’s vulnerability to suicidal behavior, including psychiatric and substance use disorders, lack of social support, family environment (for example, disrupted marital status), the occurrence of a major life stressor (for example, unemployment, grief), and various demographic factors, such as higher risk of attempted suicide among females and a greater risk of suicide completion among males [4-7]. Novel potential risk factors including biological markers, such as serum cholesterol [8], and physical measurements, such as body weight, have yet to be fully elucidated in their potential impact on suicide. The present systematic review will focus on the association between body mass index (BMI) and suicidal behavior, including completed suicide. The number of obese and overweight people worldwide has dramatically increased over the past three decades, with the number of overweight and obese adults and children rising from approximately 857 million individuals in 1980 to 2.1 billion in 2013 [9]. Excess body mass is a known risk factor of chronic illness, including cardiovascular disease, type 2 diabetes, osteoarthritis, and hypertension [10,11]. Furthermore, being overweight or obese is associated with social stigma among certain cultures, and negative attitudes towards obese individuals are evidently present in North American society [12-14]. As the prevalence of obesity rises globally [9], it is important to investigate the psychosocial burden that adiposity may have on affected individuals. Thus, an emerging body of research has attempted to investigate the relationship between BMI and suicidal behavior. Collectively, these investigations have recruited samples across all weight groups, from underweight to morbidly obese individuals. The majority of such studies have used BMI as a measure of relative body weight and as a marker of adiposity [15-21].

Considering the psychosocial stigma [14] and health related complications [10] of obesity, several systematic reviews and meta-analyses have suggested a positive association between obesity and depression [22-24]. Furthermore, about 60% of suicides occur among individuals with mood disorders [4], and 10% to 20% of patients hospitalized for depression commit suicide [25]. If obesity is indeed positively associated with depression, one might expect that a greater number of completed suicides are occurring among overweight and obese individuals. Surprisingly, however, several retrospective cohort studies have reported an inverse association between completed suicide and BMI, often in a dose-response type pattern, with the risk of suicide diminishing per unit increase in BMI [15-18,21,26]. The inverse association between suicide and BMI has been replicated in different countries using adult samples, albeit using predominantly male and Caucasian cohorts, including Swedish male conscripts [15], United States’ (US) male healthcare professionals [21], Norwegian men and women from a community cohort [18], a cohort of Danish men using conscription data [19], and a US general population cohort [17]. An American ecological study [27] reported that statewide obesity rates were inversely correlated with rates of completed suicide, and another ecological study conducted in the United Kingdom [28] similarly reported this inverse relationship in elderly females but found no such association among elderly males. Newer studies have since replicated an inverse relationship between BMI and completed suicide using longitudinal cohort designs [29,30], as well as with a case-control post-mortem investigation [31]. However, some studies have reported either no association [32-34], a J-shaped association [35], or a positive association between BMI and competed suicide [20,36]. A recent case-control post-mortem investigation [33] conducted in Australia found no association between BMI and completed suicide, failing to replicate the results of the aforementioned post-mortem design [31] out of Germany. Two recent longitudinal studies using Taiwanese [35] and German [36] cohorts reported a J-shaped and a positive relationship (respectively) between BMI and suicide. In summary, while many of the aforementioned studies point towards an inverse relationship between BMI and risk of completed suicide, the relationship remains equivocal and may depend on demographics such as sex or ethnicity. Moreover, the potential explanations for this apparent paradoxical inverse relationship remains unclear, with authors suggesting several potential mediating or correlative factors including impulsivity and case-fatality in relation to the chosen method of suicide [26,37], serum levels of cholesterol and its relation to central serotonin [8], leptin and leptin resistance [38,39], an insulin resistance based model [40], and dietary factors such as essential fatty acid intake [41].

The relationship between BMI and attempted suicide is also inconsistently reported with fewer studies compared to completed suicide, and the results are mixed [19,29,34,42-51]. Cohort studies have demonstrated a decrease in risk for attempted suicide associated with increasing BMI [19,29,34,45], while other studies have found a positive association between overweight and obese and suicide attempt [42,43,47,48,51]. Many of the studies examining BMI and attempted suicide are cross-sectional in design [42,44,46-49], thus precluding the ability to infer temporal or causal pathways. Furthermore, sex may complicate the relationship between suicide attempts and BMI, as sex was an apparent effect modifier in some reports [42]. Additionally, suicidal ideation may have a different association with body weight. Only a few studies have examined the relationship between relative body weight and suicidal ideation [42,47,48,50,52], limiting our ability to draw any conclusions. Two previous reviews have been published regarding the relationship between body weight and suicidal behavior [53,54]. One review provided a systematic review of the literature [53]; the other provided a narrative summary [54]. Both reviews cited that the collective studies were too few in number and heterogeneous to conduct a meta-analysis. Each review conducted a literature search up to 2011, and Klinitzke [53] did not include any studies published prior to January 2000. These reviews limited their searches to papers published in English, and Zhang [54] limited their search to PubMed only, precluding the inclusion of relevant articles from other databases. Zhang [54] did not present reasons for exclusion at each level of screening. Zhang reported on both adolescent and adult populations, and they classified some studies as using adult populations despite such studies including individuals as young as 15 [55,56]. The inclusion of both adolescents and adults is viewed critically due to differences in psychosocial development and differences in risk factors for suicide.

Since the conduct of these reviews, several observational studies have been published that have investigated body weight in relation to suicidal behavior [29-31,33-36,48-52], and an updated systematic review is required to contextually interpret the evidence. In addition, few studies included women in their samples when investigating BMI and suicidal behavior, and therefore, a more inclusive review will also help in understanding the association between BMI and suicidal behavior and the potential variation by sex.

### Objectives

The present systematic review aims to elucidate the association between body mass index and suicidal behavior. More specifically, the review aims to answer the following research question: Is there a significant association between body mass index (BMI) and suicidal behavior (including completed suicide, attempted suicide, suicidal ideation) in an adult population (18 years and older)? A secondary objective will be to summarize evidence for specific factors including sex, age, and the method used in suicide to assess whether such variables modify the relationship between BMI and suicidal behavior.

## Methods/design

### Inclusion and exclusion criteria

The present systematic review will include published observational studies that have reported results relating BMI to suicidal behaviors, including completed suicide, attempted suicide, and suicidal ideation, among an adult population defined as those who are 18 years or older. This adult population may include participants from population, clinical, and community-based samples. Considering that 90% of suicides occur in the context of a psychiatric disorder [4], adults from clinical samples who have a psychiatric disorder will be included as well. Other than age, no demographic limitations will be applied. Studies investigating the risk of suicide following medical treatments of obesity such as bariatric surgery or pharmacotherapy for weight loss will be excluded as the effects of medically induced weight change may affect the risk of suicidal behavior independently of BMI. The primary outcome for this review is suicidal behavior, which includes suicidal ideation, attempted suicide, and completed suicide. These outcomes are further defined in Table 1, which highlights how they were described and measured in the literature.

**Table 1:** **Definitions and statistical measures of suicidal behavior outcomes**

Outcome	Definition	Measurement of variable	Statistics	Studies
Completed suicide	Death caused by self-inflicted injurious behavior intended to end one’s own life	International classification of diseases (eighth, ninth, or tenth revisions) using diagnostic codes (E950-E950: ICD8, ICD9) or (X60-X84: ICD10)	Cox proportional hazard model	[15-21,26-28,32]
			Linear regression	
			Logistic regression	
		In some studies, associations were also estimated with undetermined deaths (deaths where it was uncertain whether the cause was suicide; (E980-E989: ICD 8, ICD 10) or codes (Y10-Y34, ICD 10))		
			Pearson correlation (ecological analysis)	
			Spearman’s rank correlation (ecological analysis)	
			Chi-square	
			Wilcoxon-Mann-Whitney test	
		Verified using mortality registries (for example, National Death Registry, National Death Index), by next of kin and/or using pathology and medical records.		
			Incidence rate ratio	
Attempted suicide	Potentially self-injurious behavior with a non-fatal outcome for which there is either explicit or implicit evidence that the person intended to kill him or herself [63].	Hospital Discharge Registries or Medical Records using aforementioned diagnostic codes for intentional injuries: (E950-E950: ICD8, ICD9) or (X60-X84: ICD10).	Cox proportional hazard model	[42-47]
				2012 to present: [29,34,48-51]
			Logistic regression	
			Incidence rate ratio	
		Self-reported using questionnaire (for example, Suicide Behaviours Questionnaire - Revised, AUDADIS, M-CIDI)		
		Self-reported using single-question (e.g. “How often have you already tried to take your own life”)		
Suicidal ideation	Having thoughts, plans, and/or impulses for suicidal behavior, including contemplation of attempting or completing suicide.	Self-reported using questionnaire (for example, Depressive Symptom Index - Suicidality Subscale, Suicide Behaviors Questionnaire - Revised).	Logistic regression Linear regression	[42,47]
				2012 to present: [48,50,52]

### Search strategy

All relevant studies will be sought and identified without any restrictions on language or time of publication. The following databases will be searched from inception: PubMed/MEDLINE, PsychINFO, EMBASE, and CINAHL. Pertinent keywords and search terms including the associated medical subject headings (MeSH) will be used in a predefined search strategy outlined in Table 2. The search will be limited to human studies. A broad search will be conducted including titles, abstracts, and keyword fields to capture studies whose title may not directly reflect the topic being investigated. Additionally, the reference lists from retrieved full-text articles and past reviews will be manually scanned for a more comprehensive search, and Cochrane review bibliographies will be hand searched. Reviews, commentaries, abstracts, and gray literature will be excluded.

**Table 2:** **Search strategy (conducted on 5 January 2015)**

Database	Search strategy
MEDLINE (*n* = 837)	1. exp Suicide/or suicid*.mp.
	2. exp Self-injurious behavior/or self-harm.mp.
	3. exp Body Mass Index/or body mass index.mp.
	4. BMI.mp.
	5. exp Body Weight/body weight.mp.
	6. exp Obesity/or obes*.mp.
	7. exp Overweight/or overweight.mp.
	8. 1 or 2
	9. 3 or 4 or 5 or 6 or 7
	10. 8 and 9
	11. limit 10 to humans
PsycINFO (*n* = 666)	1. exp Suicide/
	2. exp Attempted Suicide/or attempted suicide.mp.
	3. exp Suicidal Ideation/or suicidal ideation.mp.
	4. suicid*.mp.
	5. exp Self-Injurious Behavior/or self-harm.mp.
	6. exp Body Mass Index/or body mass index.mp.
	7. exp Body Weight/or body weight.mp.
	8. exp Obesity/or obes*.mp.
	9. BMI.mp.
	10. exp Overweight/or overweight.mp.
	11. 1 or 2 or 3 or 4 or 5
	12. 6 or 7 or 8 or 9 or 10
	13. 11 and 12
	14. limit 13 to human
EMBASE (*n* = 3688)	1. exp suicidal behavior/or suicid*.mp.
	2. exp automutilation/or self-harm*.mp.
	3. exp body mass/body mass index.mp.
	4. BMI.mp.
	5. exp body weight/body weight.mp.
	6. exp obesity/or obes*.mp.
	7. overweight.mp.
	8. 1 or 2
	9. 3 or 4 or 5 or 6 or 7
	10. 8 and 9
	11. limit 10 to human
CINAHL (*n* = 144)	1. (MH “Suicide+”) OR “suicid*”
	2. (MH “Self-Injurious Behavior”) OR “self-harm”
	3. (MH Body Mass Index) OR “body mass index”
	4. (MH “Body Weight+”) OR “body weight”
	5. (MH “Obesity+”) OR “obes*”
	6. “BMI”
	7. “overweight”
	8. 1 or 2
	9. 3 or 4 or 5 or 6 or 7
	10. 8 and 9 (limiters human)

BMI, body mass index.

### Data screening

Two independent raters (SP and RE) will screen all citations and abstracts retrieved using the search strategy outlined in Table 2, and they will subsequently identify all eligible articles according to pre-established criteria for full-text review. Discrepancies throughout the review process will be resolved by discussion and reevaluation. In cases where a consensus is not reached, a third author (ZS) will determine eligibility. Ineligible studies will be excluded from full-text review, and the reasons for exclusion will be documented. Inter-rater agreement will be calculated using the Kappa statistic [57] for each phase of screening. Authors of the included studies will be contacted if further data clarification is required.

### Data extraction

The two raters (SP and RE) will independently extract data from the studies using a premade data extraction form that will be pilot tested before screening (see Additional file 1). The information collected will include the first author and year of publication, the name of the journal, the study title, the study design, the city and country of publication, the type of the study (for example, survey, cohort), and a brief description of study participants including the number of men and women, the number of completed suicides and attempted suicides when appropriate, mean age, and ethnicity. A summary of each study’s results will be reported including the primary and secondary outcome measures, statistical analysis, and major conclusions. Authors will be contacted in the event of missing or incomplete data for clarification. If the extracted data are deemed to be suitable for a meta-analysis, individual study results will be combined to produce a summary estimate using a random-effects model as a certain degree of heterogeneity in included studies is expected.

### Risk of bias assessment

The two independent raters (SP and RE) will independently assess the risk of bias of studies included in the full-text review using the Newcastle-Ottawa scale (NOS) [58]. An adapted version of a modified NOS developed by Bawor et al. [59] will be used to assess risk of bias of observational studies included in this review (see Additional file 2). Bawor et al.’s modified NOS includes seven questions among four domains of risk assessment: methods for selecting study participants (selection bias), methods to control for confounding (performance bias), statistical methods (detection bias), and methods of exposure and outcome assessment (information bias). Risk of bias will be measured using a scale ranging from 0 (high risk of bias) to 3 (low risk of bias), and question-specific descriptions including examples of varying degrees of bias are included. Furthermore, categories pertaining to statistical methods, confounding effects, and reporting of missing data are included in this modified NOS to evaluate risk of biased methodology. Items from the original NOS pertaining to adequacy of follow-up, selection of participants (representativeness of sample), and ascertainment of outcomes, will be retained in the present adapted modified NOS, as these criteria are important to potential studies that may be included in our review.

### Statistical analysis and heterogeneity

The findings of this systematic review will be presented as a qualitative summary of the literature, and as a quantitative summary estimate, if possible. In order to determine the quality and validity of existing evidence on BMI and suicidal behavior, the results of each study will be individually and collectively assessed in terms of their study design, statistical methods, and conclusions. The *I*^2^ test statistic will be used to assess heterogeneity. An *I*^2^ value greater than 40% will be used to define substantial heterogeneity; however, regardless of the amount of heterogeneity across studies, a random-effects model will be employed when pooling the data in a meta-analysis. The random-effects model accounts for both within-study and between-study variability and it generally represents a more conservative estimate than a fixed-effects model [60]. Three separate meta-analyses will be conducted to investigate the association between BMI and completed suicide, BMI and attempted suicide, and BMI and suicidal ideation. To qualitatively assess heterogeneity, we will assess differences and similarities with respect to study samples, methods, and results. The definition of outcome may act as an additional source of heterogeneity, especially that of attempted and completed suicide (for example, deaths classified as overdoses).

As previously mentioned, women have a higher rate of attempted suicide in the general population, while men have a higher rate of completed suicide [4]. Therefore, the role of sex will be explored by stratifying results for males and females. Furthermore, it has been suggested that one’s BMI may indirectly influence one’s inclination towards a particular method of suicide, thus affecting the case-fatality of a suicidal act. For example, in a retrospective cohort study of over one million Swedish male conscripts [45], Batty et al. reported a stepwise inverse association of decreasing attempted suicide with increasing BMI. However, they noted that the strongest associations were with those methods generally requiring greater physical exertion and agility, such as hanging and jumping. These methods may be less likely to be used by those with greater BMI. Moreover, case-fatality of self-poisonings may be lowered among those with greater BMI due to greater body size and distribution of body fat [37,45]. Therefore, the role of the method used in attempted and completed suicides will be qualitatively commented on as a potential effect modifier of the relationship between BMI and attempted and completed suicide. Lastly, age will be qualitatively examined as a potential effect modifier. Medical comorbidity such as cancer that is associated with older age, weight loss, depression, or suicidal ideation, may produce a different relationship between BMI and suicidal behavior among the young and elderly [37]. Furthermore, changes in muscle mass associated with aging may result in a less accurate assessment of obesity using BMI among the elderly [21].

Review Manager 5.3 (The Cochrane Collaboration, London, UK) will be used for all statistical and pooled analyses. The results of the pooled analysis will be summarized by calculating odds ratios [ORs] or hazard ratios [HRs] and 95% confidence intervals.

### Presenting and reporting of results

**Figure 1:**
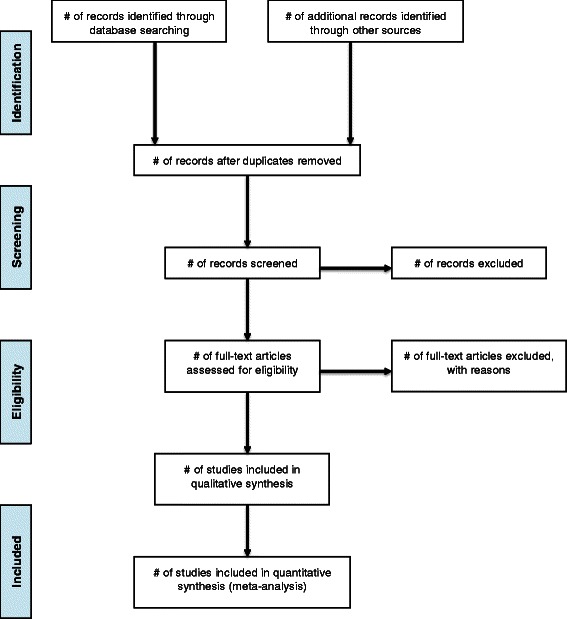
PRISMA flow diagram.

Our systematic review will be conducted and written in accordance with the preferred reporting items for systematic reviews and meta-analyses (PRISMA) guidelines [61]. A summary flow chart will be used to outline the selection of articles and to display reasons for excluding articles (Figure 1). Summary tables will be used to present the relevant outcomes and details of each included study. Egger’s plot will be used to assess potential publication bias among the selected studies.

If the studies included in our systematic review are deemed suitable for pooling in a meta-analysis, the results of each analysis will be presented in accordance with the meta-analysis of observational studies in epidemiology (MOOSE) guidelines [62]. Forrest plots will be used to present individual and pooled study estimates, and tables will be used to summarize individual study characteristics.

## Discussion

Body mass index may represent an important risk factor for suicidal behavior. The proposed systematic review will help to elucidate the association between BMI and suicidal behavior, allowing for better understanding of the controversial association between BMI and suicidal behavior. The paradox of an apparent inverse relationship between completed suicide and BMI remains to be solved. Several potential mediators, modifiers, or confounders that explain the latter association may be identified when viewing the literature systematically. BMI appears to be differentially related to completed suicide and attempted suicide, and clarifying this association may help to better understand separate etiologies that distinguish completed from attempted suicide.

## Additional files

Additional file 1:
**Data extraction form.**

Additional file 2:
**Modified Newcastle-Ottawa scale.**

## Abbreviations

BMI: Body mass index
NOS: Newcastle-Ottawa scale
