# Supplementary material for: Association between body mass index and suicidal behaviors: a systematic review protocol
Source: Syst Rev. 2015 Apr 19;4:52. doi: 10.1186/s13643-015-0038-y (PMC4424510; doi:10.1186/s13643-015-0038-y)
Supplement: Additional file 1: — Data extraction form. [file 13643_2015_38_MOESM1_ESM.pdf]

DATA EXTRACTION FORM: ASSOCIATION BETWEEN BODY MASS INDEX AND SUICIDAL BEHAVIOUR: A SYSTEMATIC REVIEW.

STUDY DETAILS

Study ID: _________ Reviewer Initials: _________

Last Name of First Author, Initial: ________________________ Year of Publication: _________

Title of Article: ____

Journal Name: ____________________________ City, Country: ____________________________

METHODS

Study Setting: ___________________________ Study Design:____________________________

Sample Size: Total __________, Men _________, Women _________

Mean Age (SD): Total ______________, Men ______________, Women ______________

Ethnicity: _____________________________________

RESULTS

Suicide Behaviour Outcome (completed suicide, attempted suicide, suicide ideation, other):
____

Outcome Definition:
____

Outcome Measurement/Assessment:
____

Number of Individuals Experiencing the event: Completed suicide ____________,
Attempted Suicide ____________

Suicide Ideation ____________, Other ____________, N/A ____________,

Statistical Testing/Methods: _____________________________________

Adjusted For:
____

Statistical Results: Coefficient ______________, 95% CI ______________, p-value ______________

Main/Additional Findings:
____

Study Limitations:
____

Inclusion Criteria (all criteria must be checked to be included):

The study examines the association between body mas index (BMI) and risk of suicidal behaviours

Study participants are ≥ 18 years of age

Study participants are human

Study has been completed and published

Exclusion Criteria (study will be excluded if any of the following are present):

Includes children and adolescents

Reports the association between risk of suicide and BMI following medical or pharmacological treatment of obesity (e.g. bariatric surgery).

Animal Study

Incomplete Study

Abstract, commentary, review

Comments:
