# Supplementary material for: Association between body mass index and suicidal behaviors: a systematic review protocol
Source: Syst Rev. 2015 Apr 19;4:52. doi: 10.1186/s13643-015-0038-y (PMC4424510; doi:10.1186/s13643-015-0038-y)
Supplement: Additional file 2: — Modified Newcastle-Ottawa scale. [file 13643_2015_38_MOESM2_ESM.pdf]

Adapted version of a modified Newcastle-Ottawa Scale for a single use in a specific context

Modified Newcastle-Ottawa Scale

Legend

0= Definitely No (high risk of bias)
1= Mostly No
2= Mostly Yes
3= Definitely Yes (low risk of bias)

Domain of evaluation: Methods for selecting study participants (*i.e. Selection Bias*)

Is the source population (cases, controls, cohorts) appropriate and representative of the population of interest?

0 (high risk of bias)	1	2	3 (low risk of bias)

Example of low risk of bias: A consecutive sample or random selection from a population that is representative of the condition under study.

Example of moderate risk of bias: A consecutive sample or random selection from a population that is not highly representative of the outcome of interest.

Example of high risk of bias: The source population cannot be defined or enumerated (i.e. volunteering or self-recruitment).

Domain of evaluation: Methods to control for confounding (i.e. Performance Bias)

Is the sample size sufficient and is there sufficient power to detect a meaningful difference in the outcome of interest?

0 (high risk of bias)	1	2	3 (low risk of bias)

Example of low risk of bias: Sample size was adequate and there was sufficient power to detect a difference in the outcome.

Example of high risk of bias: Sample size was small and there was not enough power to test the outcome of interest.

Did the study adjust for any variables or confounders that may influence the outcome?

0	1	2	3
(high risk of bias)			(low risk of bias)

Example of low risk of bias: The study identified and adjusted for all possible confounders that may influence the estimates of association between exposure and outcome.

Examples of moderate risk bias: The study identified and reported possible variables that may influence the outcome but did not statistically explore their influence.

Example of high risk of bias: The study either did not report any variables of influence or acknowledge any variables of influence when it was clear they were present.

Domain of evaluation: Statistical methods (i.e. Detection Bias)

Did the study use appropriate statistical analysis methods relative to the outcome of interest?

0	1	2	3
(high risk of bias)			(low risk of bias)

Example of low risk of bias: The study reported use of appropriate statistical analysis as required.

Examples of moderate risk bias: The study used either correct statistical methods but did not report them well, or used the incorrect methods but reported them in detail.

Example of high risk of bias: The study did not use appropriate statistical analysis as required.

Is there little missing data and did the study handle it accordingly?

0	1	2	3
(high risk of bias)			(low risk of bias)

Example of low risk of bias: The study acknowledged missing data to be less than 10% and specified the method of handling it.

Examples of moderate risk bias: The study either had greater than 15% of missing data but they specified the method used to handle it.

Example of high risk of bias: The study had greater than 15% of missing data and did not handle it at all.

Domain of evaluation: Methods of measuring outcome variables (i.e. Information bias)

Is the methodology of the outcome measurement explicitly stated and is it appropriate?

0	1	2	3
(high risk of bias)			(low risk of bias)

Example of low risk of bias: The study provides a detailed description of the outcome measure(s) which are appropriate for the outcome of interest.

Examples of moderate risk bias: The study provides a somewhat complete description of outcome measurements that are justified.

Example of high risk of bias: The study provides limited information on the methods of measuring the outcome and the measure is not appropriate considering the outcome.

Is there an objective assessment of the outcome of interest?

Example of low risk of bias: The study used objective methods to discern the outcome status of participants (*i.e. laboratory measurements, medical records*)

Examples of moderate risk bias: The study relied on subjective data as the primary method to discern the outcome status of participants (*i.e. self-report*)

Example of high risk of bias: The study had limited reporting about assessment of outcomes.

Domain of evaluation: Subject Follow-up

Was the follow-up sufficiently long enough for the outcome to occur?

0	1	2	3
(high risk of bias)			(low risk of bias)

Was there minimal loss to follow-up and are subjects lost to follow-up unlikely to introduce bias?

0	1	2	3
(high risk of bias)			(low risk of bias)

Example of low risk of bias: Follow-up was completed for all, or nearly all subjects, and reasons for losses to follow-up were well documented.

Example of moderate risk of bias: Losses to follow-up are not excessive, and reasons for

losses to follow-up are well documented and mostly unrelated to the outcome.

Example of high risk of bias: Significant loss to follow-up, reasons for losses to follow-up not reported, suspect that reasons for dropouts are related to the outcome.
